# Application of PCR in Serum Samples for Diagnosis of Paracoccidioidomycosis in the Southern Bahia-Brazil

**DOI:** 10.1371/journal.pntd.0001909

**Published:** 2012-11-29

**Authors:** Lucas Dias, Leila Falcão de Carvalho, Carla C. Romano

**Affiliations:** 1 Laboratório de Imunologia, Universidade Estadual de Santa Cruz, Ilheus, Brazil; 2 Departamento de Ciências Biológicas,Universidade Estadual de Santa Cruz, Ilheus, Brazil; Baylor College of Medicine, Texas Children's Hospital, United States of America

## Abstract

Paracoccidioidomycosis (PCM) cannot always be diagnosed by conventional means such as direct examination of histopathology or clinical samples, and serological methods, used as an alternative, still have many cases of cross-reactivity. In this scenario, molecular techniques seem to arise as a rapid approach, specific and direct that could be used in the diagnosis of this mycosis. In this study we analyzed 76 serum samples from patients in southern Bahia suspected of having paracoccidioidomycosis using a conventional PCR with primers for the ITS1 ribosomal DNA of *P. brasiliensis*. Of these 76 patients, 5 were positive for PCM by double immunodiffusion and/or direct examination and histopathology. To test specificity of PCR, we used human DNA and three isolates of *P. lutzii* (1578, 01 and ED01). Additionally, we analyzed by serial dilutions of DNA the limit of detection of the assay. The test of PCR proved specific, as only a 144 bp fragment of the three isolates of *P. lutzii* and no human DNA was amplified. Detection limit was 1.1 pg/µL of DNA. Despite the high detection limit and specificity of PCR none of the 76 serum samples were found positive by PCR, but a biopsy specimen obtained from one of the patients with PCM was positive. These results, albeit limited, show that PCR is not effective in detecting DNA of *P. brasiliensis* or *P. lutzii* in serum, but could perhaps be used with other types of clinical samples, especially in those instances in which conventional methods fail.

## Author Summary

Paracoccidioidomycosis (PCM) has been included in such diseases neglected since this impact on public health have not been measured. Except in the South and Southeast of Brazil, there are no government programs for this mycosis. After 100 years of discovery of the disease there is the need to deploy an effective and continuous program for the prevention, diagnosis and treatment. PCM requires a prolonged treatment, generally greater than 1 year. Moreover, the patients with PCM are associated with other co morbidities such as alcoholism, smoking and malnutrition.

To aggravate this scenario the serological diagnosis of PCM in many cases results in cross reactivity with other mycoses. Thus, this study aimed to develop a PCR which can be used in association with the serological techniques to improve the diagnostic these individuals. We work with plasma samples from patients in northeastern Brazil, in an area where the disease has not been reported. None of the 76 plasma samples were positive by PCR, but a biopsy specimen obtained from a patient was positive. These results reinforce that PCR have the limitation when serum or plasma is used. However, PCR can be an important diagnostic tool when conventional diagnostic methods are not successful.

## Introduction

Paracoccidioidomycosis (PCM) is a deep mycosis caused by the thermo-dimorphic fungus *Paracoccidioides brasiliensis*, endemic in some countries of Latin America, mainly in Brazil [1]. In this disease, the fungus can remain confined in the lungs, the primary focus of infection, or spread to other organs and tissues, resulting in different clinical manifestations. The form acute and sub acute occurs in young people of both sexes, mainly affecting the reticuloendothelial system. The chronic form of PCM predominates in adult males and is characterized by presence of the fungus restricted in the lungs and/or disseminated to the mucosa, skin and lymph nodes [2].

The conventional diagnosis of PCM is based on viewing and/or isolation of the fungus in clinical specimens. However, the specimen may not always be viewed and microbiological culture is time-consuming and mostly negative [3]. Serological techniques have been employed, but there are still many cases of cross-reactivity with other fungal species [4]. In addition, false-negative results can be obtained in cases where the patient has some type of immunodeficiency [5]. Nevertheless, the scene of the PCM have been changed since the discovery of a new species, *P. lutzii*, which has very distinct behaviors of *P. brasiliensis* [6].

Therefore, a molecular approach appears to be an excellent alternative in the diagnosis of PCM. However, so far this technique has not been used routinely in the diagnosis of PCM. To this end specific primers for conserved regions have been developed, i.e., for detection of 18S, 5.8S and 28S rDNA and their regions ITS1 and ITS2, of the gp43 gene and an antigenic protein of 27 kDa [7], [8], [9], [10]. Although the gp43 gene is one of genes used in the classification of cryptic species [11] and some studies show a relationship between polymorphisms of these gene and pathogenicity [9], we believe that perhaps not all isolates of *P. brasiliensis or P. lutzii* have the gp43 gene.

On the other hand, ribosomal DNA is present in all isolates and there are conserved regions within this structure, thus primers for these regions would be more appropriate in terms of sensitivity and specificity [12]. Thus, we aim to develop a conventional PCR using a pair of primers specific for the known ITS1 region of ribosomal DNA of *P. brasiliensis* [13], and know what is the viability of this PCR in serum samples. We also made an effort to determine whether this PCR assay could be used to detect DNA of *P. lutzii*.

## Materials and Methods

### Isolates of *P. lutzii*

We use three isolates of *P. lutzii* (1578, 01 and ED01) gently provided by Professor Carlos Taborda from the Laboratory of Pathogenic Dimorphic Fungi from the Institute of Biomedical Sciences II of Universidade de São Paulo (Sao Paulo, Brazil).

### DNA extraction of isolates

DNA of the three isolates was extracted as described by Kennedy et al. [14], with some modifications. Briefly, 0.4 g yeast was macerated with liquid nitrogen and the resulting powder was transferred to a 2 mL microtube. Then 1.7 mL of extraction buffer (100 mM EDTA, 100 mM Tris, 1.5 M NaCl, 1% SDS, 2% CTAB) were added and the mixture was incubated for 20 min at 65°C (inverting the microtube every 5 min) before centrifugation (20 min at 4500× g, Centrifuge MiniSpin, Eppendorf-AG, Germany). The supernatant was collected and transferred to a new microtube. An equal volume of phenol-chloroform-isoamyl alcohol (25∶24∶1) was added, and after homogenization the tube's content was centrifuged at 4500× g for 10 min. The supernatant was collected and 0.7 volume of isopropanol (100%) and 0.1 volume of 3 M sodium acetate were added and the content mixed by gently inverting the microtube 10 times. After overnight storage at −20°C the sample was centrifuged for 10 min at 4500× g and the resulting pellet washed twice with 70% ethanol. The pellet was dried, resuspended in 200 µL MilliQ water and analyzed by electrophoresis in 2% agarose gel in TBE buffer (40 mM Tris base, 20 mM boric acid, 1 mM EDTA) at 100 V for 30 min and GelRed staining (Uniscience of Brazil, Brazil). Quantification was performed in GeneQuant *pro* (Amersham Bioscience, USA).

### Primers

We used the primer pair described by Buitrago et al. [13] in a trial of Real-time PCR, but adapted for a classic PCR and using *P. lutzii* not *P. brasiliensis*. The direct primer (OliPbMB1) was 5′-ACCCTTGTCTATTCTACC-3′ and reverse primer (OliPbMB2) was 5′-TTACTGATTATGATAGGTCTC-3′, which generated a 144 bp fragment amplified from the region ITS1 of the rDNA of *P. brasiliensis*. Primers were synthesized by the Bioneer Corporation (CA, USA).

### PCR

The reaction was performed in 0.2 mL sterile microtube containing 10 ng DNA, 75 mM tris-HCl (pH 8,8), 20 mM (NH_4_)_2_SO_4_, 1.5 mM MgCl_2_, 1.5 mM dNTP (Fermentas Inc., MA, USA), 0.4 pM of each primer, 1 U Taq polymerase (Fermentas Inc., MA, USA) and sterile milli-Q water, obtaining a final volume of 25 µL. The reactions were processed in the Veritas thermal cycler (Applied Biosystems, CA, USA) programmed as follows: 96°C for 5 min; 40 cycles of 55°C for 35 s, 72°C for 35 s and 96°C for 35 s; 72°C for 7 min. Positive control was DNA extracted from *P. lutzii* (isolate 01) and negative control was sterile water milli-Q. To visualize the reaction, 5 µL of the amplified product was applied in agarose gel 2% in TBE buffer, at 100 V for 1 h and stained with GelRed.

### Detection limit and assay of specificity

Detection limit of the PCR assay was established by using serial dilutions of *P. lutzii* DNA (from 120 ng/µL to 0.5 pg/µL) and specificity was evaluated with DNA from humans and from the isolates of *P. lutzii* (1578,01 e ED01).

### Clinical samples

We analyzed serum samples from 76 patients (citizens from Itabuna and Ilhéus), suspected to have PCM and no conclusive diagnosis to another fungal disease/non-fungal. Five of these patients had been diagnosed with PCM in our laboratory by the method of double immunodiffusion alone (5) or in combination with direct examination of sputum and/or histopathology (3). Furthermore, the diagnosis was endorsed by a clinical experience of the physician responsible for the patient. These five patients had the chronic form of the disease and had not started treatment before blood collection. Only one of these had associated tuberculosis/paracoccidioidomycosis. The care and clinical monitoring of these patients was conducted in city of Itabuna at the Center for Health José Maria de Magalhaes or Santa Casa de Misericordia or at the Specialized Care Center III, located in Ilhéus.

### Ethics statement

All individuals/patients were informed about the methodology and signed an informed consent according to ethical standards. The project was also approved by the Ethics in Research Committee of the Universidade Estadual de Santa Cruz (Text S1).

### DNA extraction of clinical samples

DNA extraction from serum samples used QAamp DNA Mini Kit (Qiagen, Hilden, Germany). We used 200 µL of sample and smaller volumes were adjusted with phosphate buffer (PBS, pH 7.4). Purified DNA was eluted in 25 µL of elution buffer and analyzed by electrophoresis in 2% agarose gel in TBE buffer at 100 V for 30 min (stained with GelRed). Quantification of extracted DNA was performed in GeneQuant *pro*. We also extracted DNA from a biopsy sample of one the patients with confirmed PCM diagnosis. All DNA samples were then stored at −20°C until use in assay of PCR.

### Double immunodiffusion

The test was performed in mesh citrate-agarose (1% agarose, 0.4% sodium citrate, 0.9% sodium chloride and 7.5% glycine). In the central well apply 10 µL of exoantigen of *P. brasiliensis*, 339 isolated, and the side wells 10 µL of serum from patients or healthy subjects. Slides were incubated in a humid chamber at room temperature for 48 hours, with readings every 24 hours. Subsequently, they were washed with sodium citrate to 5% and 0.85% physiological solution, dried at 60°C for 24 hours and stained with coomasie brilliant blue R-250 0.15% (Vetec, Rio de Janeiro, Brazil) in water-methanol-acetic acid (4∶4∶1) for 5 minutes. Positive samples were serially diluted (1/2 to 1/256) and re-subjected to the test ID for semiquantitative analysis. As a positive control we used the reaction hyperimmune serum of rabbit anti-exoantigens of *P. brasiliensis*, obtained from the Immunodiagnostic Laboratory of Mycoses of the Adolfo Lutz Institute (São Paulo) and kindly provided by Dr. Adriana Vicentini Pardini.

## Results

### Validation of PCR with DNA of isolates of *P. brasiliensis* and *P. lutzii*

**Figure 1:**
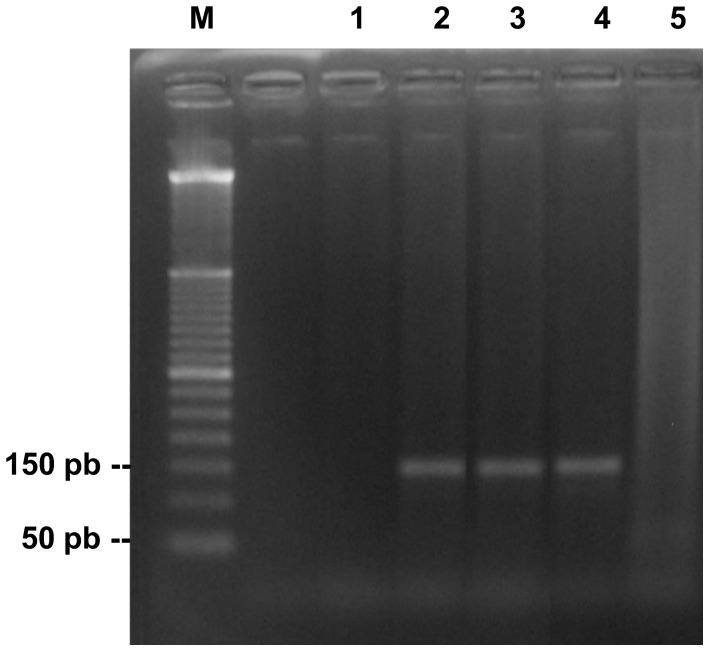
PCR assay revealing the target fragment of 144 pb in the DNA of *P. lutzii*. M, molecular marker of 50 bp; 1, negative control; 2, 3 and 4, DNA of *P. lutzii* isolates 01, ED01 and 1578, respectively; 5, human DNA.

The functionality and specificity of PCR standardized by us can be proven by visualization of the 144 pb amplicon generated when using DNA from isolates of *P. lutzii* (Fig. 1). The reaction with *P. brasiliensis* (isolate 03) also generated the same amplicon (Fig. S1). Human DNA served as specificity control and was always negative.

### Detection limits

**Figure 2:**
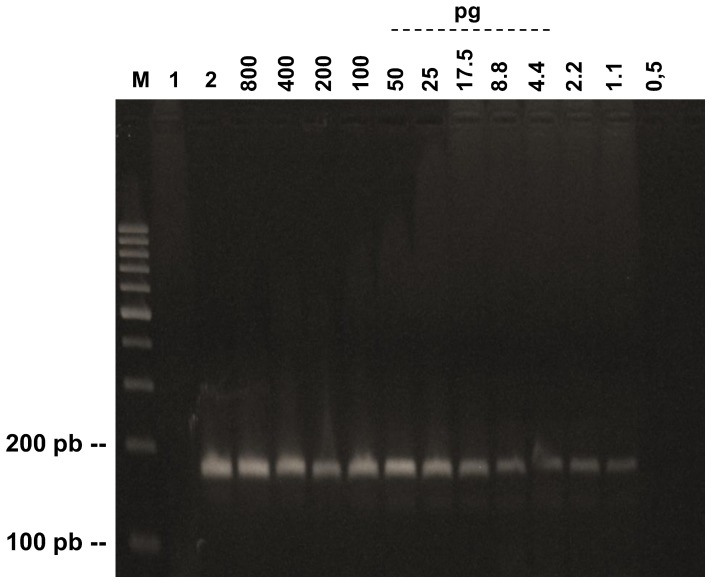
Detection limit of PCR assay with different amounts of DNA of *P. lutzii*. M, 100 bp DNA ladder; 1, negative control; 2, positive control.

Our standardized PCR test was able to detect until 1.1 pg of DNA of *P. lutzii* (Fig. 2). The range of detection of the test was from 1.1 pg/µL to 60 ng/µL of DNA.

### Application of PCR in serum samples

**Figure 3:**
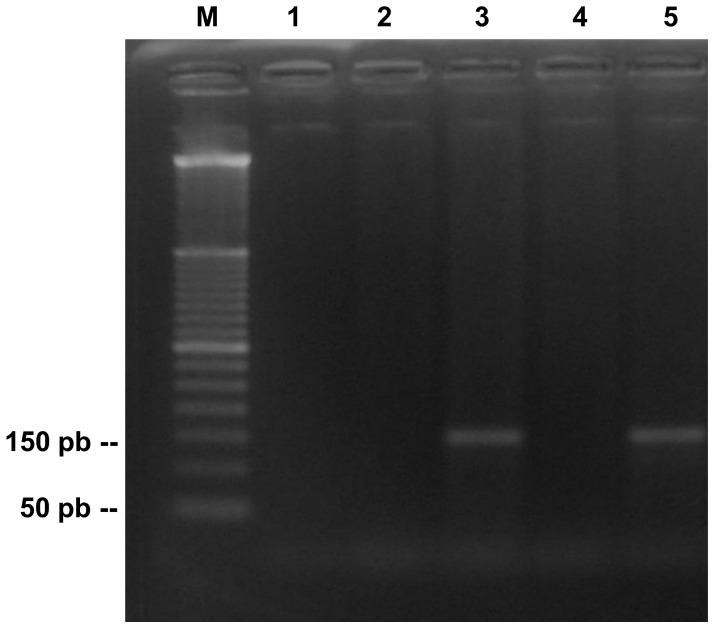
Analysis of clinical samples (serum and biopsy) of PCM by PCR. Lane M, 50 bp DNA ladder; lanes 1 and 2, samples of serum; lane 3, biopsy specimen; lane 4 e 5, negative control and positive, respectively.

We analyzed seventy-six serum samples of patients with suspected PCM. Five of these patients has been diagnosed with PCM by double immunodifusion (Fig. S2), and three of them were confirmed by direct examination of sputum or histopathology (Fig. S3). In the PCR, none of the seventy-six serum specimens were positive. However, the single biopsy specimen that was tested was positive, revealing the expected 144 pb fragment (Fig. 3).

## Discussion

In those cases where the microscopic observation of *P. brasiliensis* is not successful and when the levels of antigen or antibody are very low, the use of molecular techniques such as PCR has been shown to be effective [15]. There is much antigenic variation among isolates of *P. brasiliensis* from the different parts of Brazil and Latin America, and as consequence differences in serologic reactivity have been observed, showing the need to work with antigens specific for isolates from a defined region [16], [17].

Considering these aspects and the fact that we use antigens of isolates from other regions in the double immunodiffusion test to detect cases of PCM in Southern Bahia and that we have not yet succeeded in isolating the fungus, the use of PCR in detection of PCM seemed more appropriate.

For these purpose, the primers pairs specific for the ITS1 region of rDNA of *P. brasiliensis* described by Buitrago et al. [13] seemed more suitable for application of PCR as a diagnostic tool. Therefore, this primes pairs allowed the detection of both DNA *P. lutzii* as *P. brasiliensis*, being generated the same 144 pb amplicon. However, because to this characteristic, it is not possible to distinguish which of the two species is the DNA amplified from single biopsy sample tested.

Our results reinforce the fact that PCR and PCR-based molecular techniques have a limitation when serum or plasma is used [18], [19]. This can be explained because of the fungus is rarely in the blood stream [20]. Another possibility is that in this body part the yeast and DNA of *P. brasiliensis* is readily phagocytosed by leukocytes, reducing the chances of finding these elements there [21].

Despite these issues, we conducted an experiment where we evaluated the possibility of directly using the serum/plasma, without extraction of DNA, in the PCR reaction (data not shown). In this case, serum/plasma could contain the yeast *P. brasiliensis* or *P. lutzii* and/or its DNA dispersed, or none at all of these elements. Considering the case of the yeast to be integrated, we mixed a plasma sample a predetermined amount of the yeast *P. brasiliensis* and submitted to the PCR reaction but the result was negative.

Interestingly, when conducting another test with a plasma sample received 10 ng of DNA of *P. brasiliensis*, the reaction was positive. This experiment leads us to two hypothesis: that in plasma not exist inhibitory elements of the PCR reaction or that these elements exist but are at low levels and are further diluted when mixed with other components of the PCR reaction mix. More tests are needed to confirm this finding to be applied in the PCR reactions in general.

In summary, we include some information about the limitations (Text S2) and experimental design of this study (Fig. S4). We believe that PCR can be used as a diagnostic tool in diagnosis of PCM, especially when conventional diagnostic methods are not successful, but much remains to be done to make this molecular test secure. Moreover, we know that quantitative molecular methods, such as real-time PCR, would be more appropriate to predict disease or infection. However, this is an expensive technique and would not be performed at some locations. Thus, more studies are needed that aim to refine and increase the discriminatory power of more feasible molecular techniques as conventional PCR or nested-PCR.

## Supporting Information

Figure S1 **The primer pair OliPbMB1/OliPbMB2 generate fragments of the same size both in** ***P. brasiliensis*** **as in** ***P. lutzii*** **.** M, molecular marker of 100 bp; 1, negative control; 2 and 3, DNA of *P. lutzii* (isolate 01) and *P. brasiliensis* (isolate 03), respectively.
(TIF)

Figure S2 **Double immunodiffusion test revealed antibody anti-** ***P. brasiliensis*** **in the sera of five patients(P1–5).** The slides were stained with Coomasie Brilliant Blue R-250 0.15%. Ag: exoantigens of *P. brasiliensis*, isolate 339; C+: rabbit serum anti-exoantigens of *P. brasiliensis*.
(TIF)

Figure S3 **Three patients positive by immunodiffusion were confirmed by direct examination or histopathology.** The arrow show *P. brasiliensis* in multiple budding in direct examination (A) and as yeast single in histopatology (B). Direct examination was performed on sputum samples and using 20% KOH solution as a clarifier. Histopathology was performed only in one patient, from a liver biopsy and stained with hematoxylin and eosin. in this photo, 400× magnification.
(TIF)

Figure S4 **Flowchart showing the method of experimental analysis of patient samples submitted to the study.**
(TIF)

Text S1 **Approval of the Ethics Committee in Research of Universidade Estadual de Santa Cruz.**
(DOCX)

Text S2 **STARD Checklist.**
(DOC)
