# Supplementary figures and images for: Application of PCR in Serum Samples for Diagnosis of Paracoccidioidomycosis in the Southern Bahia-Brazil
Source: PLoS Negl Trop Dis. 2012 Nov 29;6(11):e1909. doi: 10.1371/journal.pntd.0001909 (PMC3510084; doi:10.1371/journal.pntd.0001909)

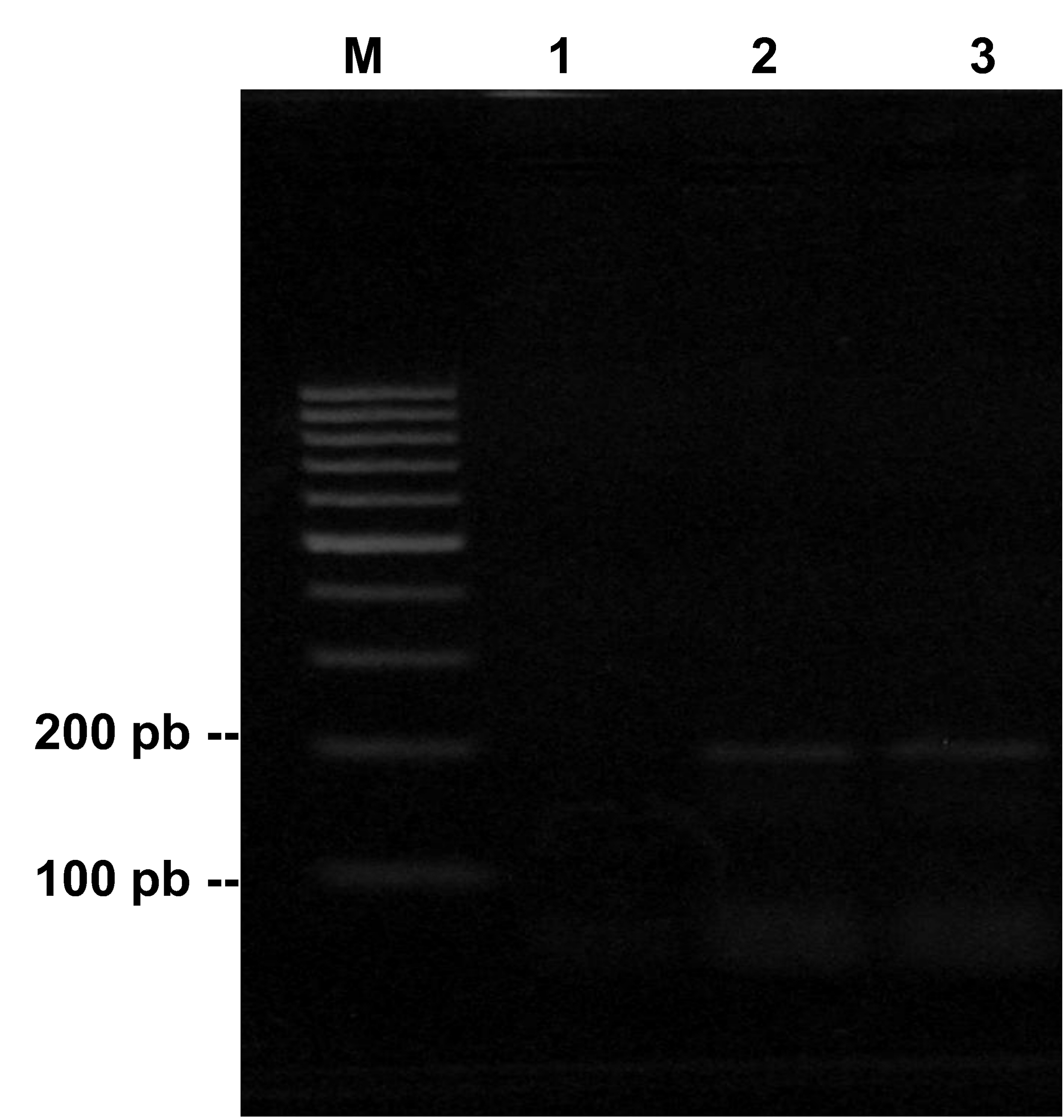

Supplement: Figure S1 — The primer pair OliPbMB1/OliPbMB2 generate fragments of the same size both in P. brasiliensis as in P. lutzii . M, molecular marker of 100 bp; 1, negative control; 2 and 3, DNA of P. lutzii (isolate 01) and P. brasiliensis (isolate 03), respectively. (TIF) [file pntd.0001909.s001.tif]

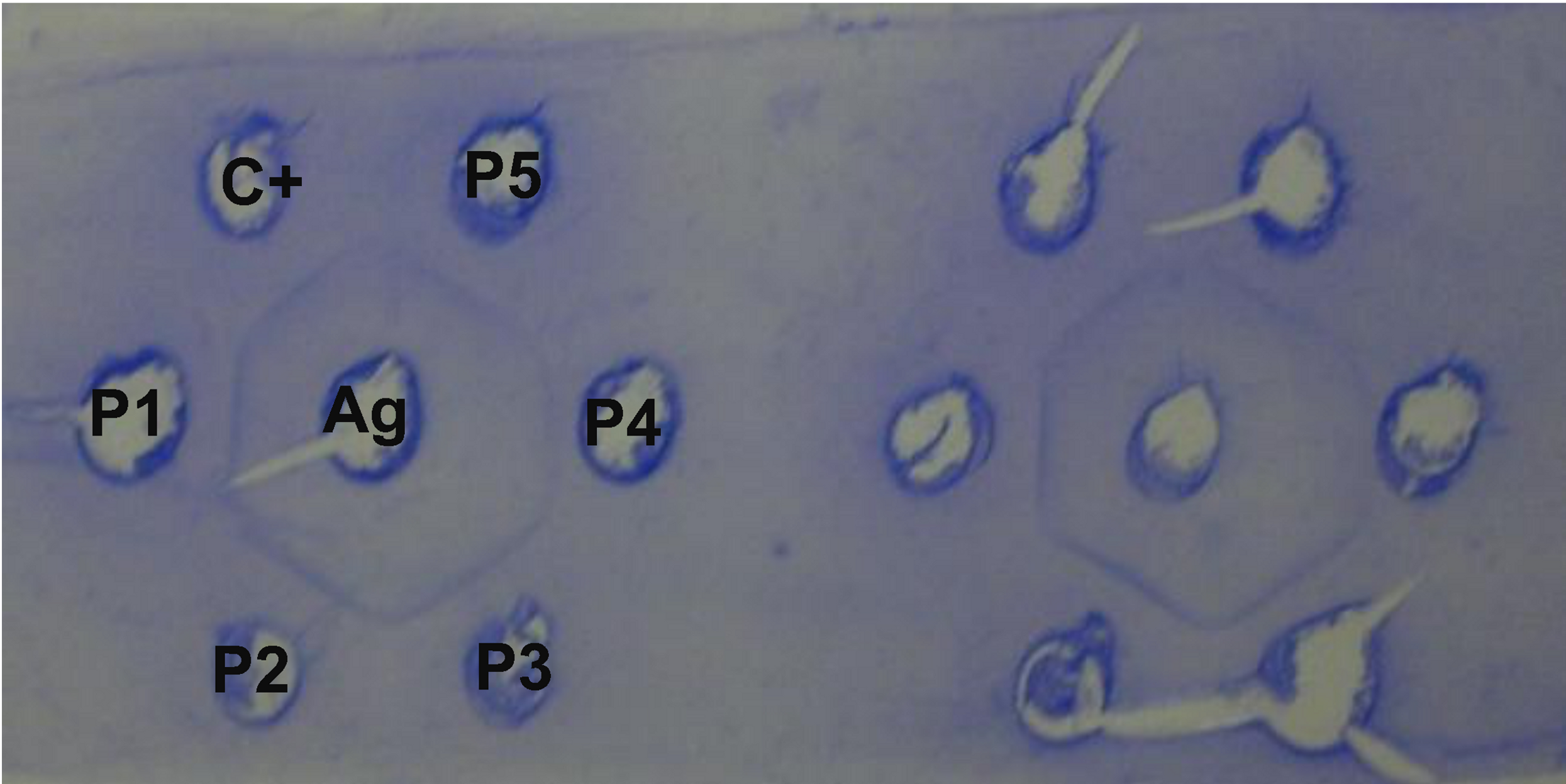

Supplement: Figure S2 — Double immunodiffusion test revealed antibody anti- P. brasiliensis in the sera of five patients(P1–5). The slides were stained with Coomasie Brilliant Blue R-250 0.15%. Ag: exoantigens of P. brasiliensis, isolate 339; C+: rabbit serum anti-exoantigens of P. brasiliensis. (TIF) [file pntd.0001909.s002.tif]

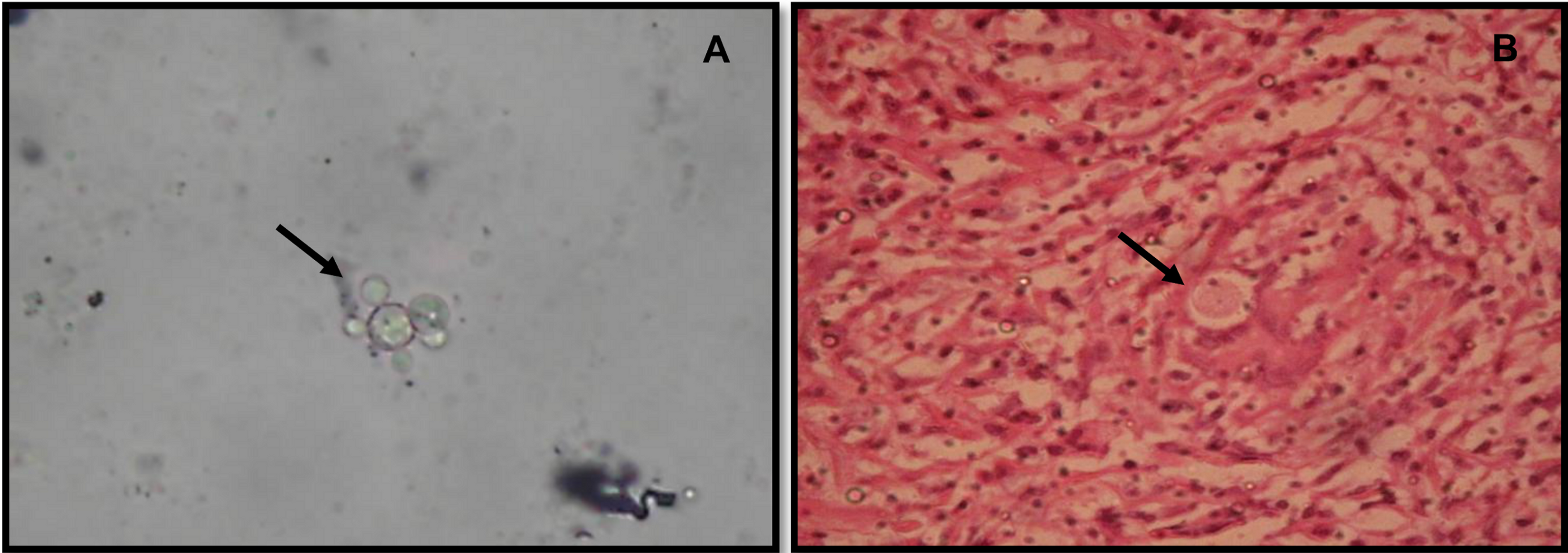

Supplement: Figure S3 — Three patients positive by immunodiffusion were confirmed by direct examination or histopathology. The arrow show P. brasiliensis in multiple budding in direct examination (A) and as yeast single in histopatology (B). Direct examination was performed on sputum samples and using 20% KOH solution as a clarifier. Histopathology was performed only in one patient, from a liver biopsy and stained with hematoxylin and eosin. in this photo, 400× magnification. (TIF) [file pntd.0001909.s003.tif]

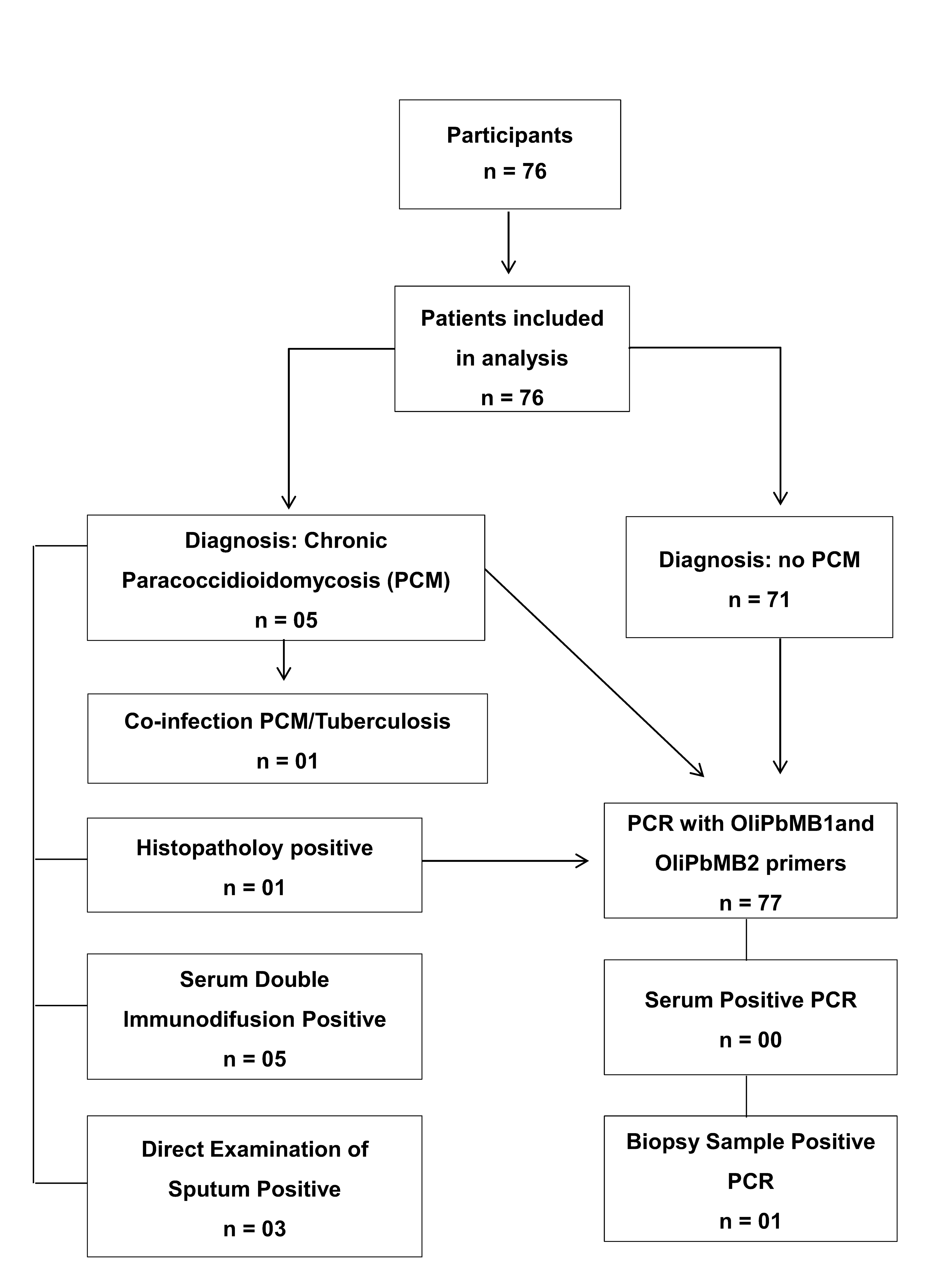

Supplement: Figure S4 — Flowchart showing the method of experimental analysis of patient samples submitted to the study. (TIF) [file pntd.0001909.s004.tif]
