# Supplementary material for: Application of PCR in Serum Samples for Diagnosis of Paracoccidioidomycosis in the Southern Bahia-Brazil
Source: PLoS Negl Trop Dis. 2012 Nov 29;6(11):e1909. doi: 10.1371/journal.pntd.0001909 (PMC3510084; doi:10.1371/journal.pntd.0001909)
Supplement: Text S1 — Approval of the Ethics Committee in Research of Universidade Estadual de Santa Cruz. (DOCX) [file pntd.0001909.s005.docx]

UNIVERSIDADE ESTADUAL DE SANTA CRUZ


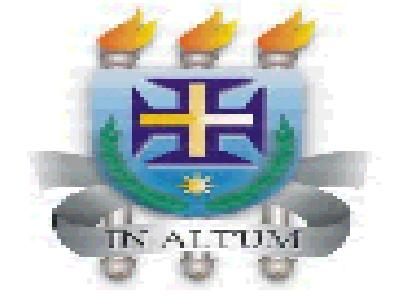


**UESC**

**ETHICS COMMITTEE IN RESEARCH - CEP/UESC**

# **The consolidated No. 296**

# -Regular Meeting No. 75, 23/Julho/2008-

**Protocol**: 195/08

**Responsible researcher:**CARLA CRISTINA ROMANO

**Project Title:**"Interaction of *Paracoccidioides brasiliensis* isolate (Pb339) with human mononuclear cells: mechanisms of pathogenicity and immune activation."

The research protocol will produce scientific knowledge about the interaction of an antigen isolated from *Paracoccidioides brasiliensis* (Pb339) with human mononuclear cells, as well as carry out a serological survey in the cities of Ilheus and Itabuna to elucidate the incidence of infection and disease due to establishment of pulmonary mycosis.

The scientific relevance of this study is justified by the lack of research on the immunological profile of the population in the cities mentioned above, as well as the results of this study may lead to an immediate therapeutic management of infected people.

The project presents appropriately the scientific problem, the objectives, the theoretical framework, the literature review and methodological procedures. After presentation and analysis by this Committee, with due return by the researcher of disputes and questions raised (the consolidated No. 288), it was found that the research protocol meets the requirements of Resolution CNS n º 196/96.

**Status of the Protocol**: Given the favorable relationship "benefits / risks", and there was no elements that constitutes the ethical commitment in achieving the project, it was considered **APPROVED** by this Committee.

According to Resolution CNS 196/96, partial and final reports as well as possible methodological changes during the execution of the work should be reported and sent to the CEP-UESC for follow up. Importantly, the supplier (s) researcher (s) on the consequences of the research does not end with the completion of the data collection stage, but extends beyond the end of the research project, to the stage dissemination and application of results.

Campus Soane Nazaré de Andrade, 4 of August of 2008.
